# Chrysin attenuates diabetes‐induced retinal injury by inhibiting microglial GBP3/NLRP3/GSDMD‐mediated pyroptosis

**DOI:** 10.1002/ame2.70293

**Published:** 2026-09-24

**Authors:** Qun Liu, Song‐min Wang, Xuan Chen, Yi‐rao You, Chen‐jiao Guo, Chang‐qin Xie, Lu‐lu Wang, Qi Liu, Lan Zhang, Jun‐tao Yang

**Affiliations:** ^1^ School of Basic Medicine Nanchang Medical College Nanchang PR China; ^2^ Innovative Research Center for Major Disease Mechanisms and Intervention Nanchang Medical College Nanchang PR China; ^3^ School of Nursing Guangdong Pharmaceutical University Guangzhou PR China; ^4^ Shenzhen Binhai Aier Eye Hospital Shenzhen PR China; ^5^ Department of Ophthalmology The Second Affiliated Hospital, Jiangxi Medical College, Nanchang University Nanchang PR China; ^6^ Chinese Academy of Medical Sciences & Peking Union Medical College Beijing PR China

**Keywords:** chrysin, diabetic retinopathy, GBP3, microglia, pyroptosis

## Abstract

**Background:**

This study was designed to investigate whether chrysin, a natural flavone, alleviates experimental diabetic retinal disease and to define a microglia‐centered mechanism involving guanylate‐binding protein 3 (GBP3) and NLR family pyrin domain containing 3 (NLRP3)–gasdermin D (GSDMD) pyroptosis signaling.

**Methods:**

Streptozotocin (STZ)‐diabetic mice received vehicle or chrysin for 4 weeks, followed by assessment of fasting blood glucose, serum caspase‐1 and interleukin‐1β (IL‐1β) levels, retinal histology, Evans blue leakage, and retinal immunofluorescence for glial fibrillary acidic protein (GFAP) and ionized calcium‐binding adaptor molecule 1 (IBA‐1), with apoptosis measured by terminal deoxynucleotidyl transferase dUTP nick end labeling (TUNEL). In vitro, BV2 microglia were exposed to high glucose with vehicle or chrysin. Cell viability (Cell Counting Kit‐8), oxidative stress (reactive oxygen species and malondialdehyde), and pyroptosis‐associated markers were analyzed by Western blotting, reverse transcription quantitative polymerase chain reaction (RT‐qPCR), and immunofluorescence. RNA sequencing (RNA‐seq) profiled transcriptional responses, and small interfering RNA (siRNA) was used to knock down GBP3.

**Results:**

In STZ‐diabetic mice, chrysin reduced fasting glucose and circulating caspase‐1 and IL‐1β, partially preserved retinal thickness, and decreased vascular leakage. Chrysin attenuated GFAP upregulation, reduced IBA‐1 signals, and lowered TUNEL‐positive cells. In BV2 cells, chrysin improved viability, reduced oxidative stress, and suppressed NLRP3–GSDMD activation. RNA‐seq highlighted chrysin‐responsive pyroptosis signatures and identified GBP3 as a regulated node. GBP3 knockdown reduced high‐glucose‐associated NLRP3 and Gsdmd expression and diminished GSDMD cleavage.

**Conclusions:**

Chrysin protects the diabetic retina and suppresses microglial pyroptosis via a GBP3‐linked NLRP3–GSDMD axis.

## Graphical abstract

We investigated the therapeutic potential and mechanism of chrysin in experimental diabetic retinal disease. Using a streptozotocin (STZ)‐induced diabetic mouse model and high‐glucose‐stimulated BV2 microglia, we revealed chrysin‐responsive pyroptosis‐associated signatures and identified GBP3 as a regulated node. These findings show that chrysin reduced inflammasome/pyroptosis‐related markers in diabetic retinas.

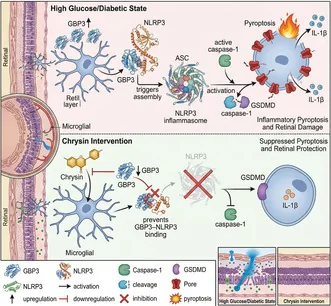

## 1 INTRODUCTION

Diabetic retinopathy (DR) remains a leading cause of preventable vision loss among working‐age adults worldwide, imposing substantial personal and societal burdens.^1^, ^2^, ^3^ With ~30% of people with diabetes developing some degree of DR and >103 million individuals currently affected, the rising global prevalence of diabetes—projected to exceed one billion by 2050—portends a further escalation in DR‐related morbidity and costs.^2^, ^4^, ^5^ Clinically, DR is classically staged as nonproliferative (mild, moderate, severe) and proliferative disease, reflecting a spectrum of microvascular injury that includes microaneurysms, intraretinal hemorrhages, capillary nonperfusion, venous abnormalities, intraretinal microvascular abnormalities (IRMA), and, at advanced stages, pathologic neovascularization; diabetic macular edema arising from increased vasopermeability is a major cause of central vision loss.^2^, ^6^, ^7^ Although historically viewed as a microvascular disorder, accumulating evidence indicates that retinal neurodegeneration precedes and potentially drives microvascular pathology, leading to a broader construct termed “diabetic retinal disease” that captures neuronal and glial involvement alongside vascular changes.^6^, ^7^

Within this neurovascular framework, retinal glia—principally Müller cells, astrocytes, and resident microglia—have emerged as critical modulators of diabetic retinal injury. Chronic hyperglycemia and hypoxia drive inflammatory and oxidative stress pathways, including NLRP3–inflammasome activation in macroglia and microglia, leading to upregulation of IL‐1β, IL‐6, ICAM‐1, and VEGF and thereby linking glial activation to proangiogenic signaling.^2^, ^8^ In parallel, oxidative stress‐induced AP‐1/NF‐κB activation promotes caspase‐dependent neuronal apoptosis accompanied by Müller and astrocytic reactive gliosis, which is characterized by increased GFAP expression and is detectable even in diabetic eyes without clinically apparent DR.^9^ Mitochondrial dysfunction within Müller glia further amplifies reactive oxygen and nitrogen species, fueling microglial activation and bursts of oxidative stress that damage the neurovascular unit and promote focal retinopathic lesions.^4^ Together, these findings indicate that glial–microglial signaling is a key driver of diabetic retinal disease and justify glia‐targeted antioxidant and anti‐inflammatory approaches as adjuncts to vascular‐directed therapy.

Building on the concept of glia–microglia‐driven neuroinflammation, naturally occurring flavonoids have attracted interest as pleiotropic modulators of oxidative stress and immune signaling. Chrysin (5,7‐dihydroxyflavone), a flavonoid found abundantly in propolis and *Scutellaria baicalensis*, exerts potent antioxidant, anti‐inflammatory, and neuroprotective actions in the central nervous and immune systems that have been investigated in ischemic stroke and other neurological disorders.^10^, ^11^, ^12^ In this study, we therefore sought to elucidate the mechanistic effects of chrysin in experimental DR, with a specific focus on its impact on retinal microglia as key local immune effector cells within the neurovascular unit.

## 2 METHODS

### 2.1 Chemicals and reagents

BV2 microglial cells were purchased from Procell Life Science & Technology Co., Ltd. (Wuhan, China). Chrysin (purity 99.85%, HY‐14589) was obtained from MedChemExpress (Shanghai, China). Primary antibodies against GFAP (16825‐1‐AP), Iba‐1 (10904‐1‐AP), caspase‐1 (31020‐1‐AP), IL‐1β (16806‐1‐AP), NLRP3 (30109‐1‐AP), ASC (10500‐1‐AP), and GSDMD (20770‐1‐AP) were purchased from Proteintech (Wuhan, China). An additional GSDMD antibody (AF4012) was obtained from Affinity Biosciences (Jiangsu, China). HRP‐linked secondary antibodies, including anti‐mouse IgG (550108) and anti‐rabbit IgG (550018), as well as goat anti‐rabbit Alexa Fluor 594 (550043) and goat anti‐mouse Alexa Fluor 488 (550036), were supplied by Zenbio (Chengdu, China). Cell Counting Kit‐8 (C0037), Hoechst/PI staining buffer (C0003), reactive oxygen species (ROS) assay kit (S0035S), and malondialdehyde (MDA) assay kit (S0131S) were purchased from Beyotime Biotechnology (Shanghai, China). Mouse IL‐1β enzyme‐linked immunosorbent assay (ELISA) kits (MU30369) were obtained from Bioswamp Biotech (Wuhan, China). All other reagents, unless otherwise specified, were purchased from Sigma‐Aldrich (St Louis, MO, USA).

### 2.2 Cell culture and treatment

Murine BV2 microglial cells were maintained in complete Dulbecco's Modified Eagle's Medium (DMEM; Procell, Wuhan, China) supplemented with 5% fetal bovine serum (FBS) and 1% penicillin–streptomycin. Cells were cultured at 37°C in a humidified incubator with 5% CO_2_. To establish a high‐glucose model, BV2 cells were first exposed to DMEM containing 30 mmol/L D‐glucose for 24 h, followed by continuous treatment with 10 μmol/L chrysin for an additional 24 h in the same high‐glucose medium.

### 2.3 Cell viability and MDA assay

Cell viability was assessed using the CCK‐8 assay. BV2 cells were seeded into 96‐well plates, exposed to the indicated treatments, and then incubated with 10% CCK‐8 working solution for 2 h at 37°C; absorbance at 450 nm was measured and viability was expressed as a percentage of the control. Lipid peroxidation was evaluated by measuring malondialdehyde (MDA) levels using an MDA assay kit; after treatment, cells were lysed and processed according to the manufacturer's instructions, absorbance at 532 nm was recorded, and MDA content was calculated from a standard curve, normalized to protein concentration, and expressed as nmol/mg protein.

### 2.4 ROS assay and Hoechst/PI staining

ROS were measured using CM‐H₂DCFDA. BV2 cells cultured in 48‐well plates were washed with PBS and incubated with 5 μmol/L CM‐H₂DCFDA for 30 min at 37°C in the dark, and fluorescence was then recorded using an inverted fluorescence microscope. Nuclear morphology and cell death were assessed by Hoechst 33342/propidium iodide (PI) double staining. After treatment, cells were incubated with the Hoechst/PI staining solution according to the manufacturer's instructions, gently washed with PBS, and observed by fluorescence microscopy, with PI‐positive nuclei considered non‐viable.

### 2.5 Western blot

BV2 cells and retinal tissues were lysed in ice‐cold RIPA buffer supplemented with protease and phosphatase inhibitors, and protein concentrations were determined using a BCA protein assay kit. Equal amounts of protein (20–30 μg per lane) were separated on 10%–12% SDS–polyacrylamide gels and electrophoretically transferred onto PVDF membranes. After blocking with 5% bovine serum albumin in TBST for 1–2 h at room temperature, membranes were incubated overnight at 4°C with the indicated primary antibodies. The following day, membranes were washed, incubated with HRP‐conjugated secondary antibodies, and visualized using an enhanced chemiluminescence (ECL) detection system.

### 2.6 RT‐qPCR

Total RNA was extracted from BV2 cells and retinal tissues using TRIzol reagent according to the manufacturer's instructions. After assessing RNA purity and concentration, 0.5–1.0 μg of RNA was reverse‐transcribed into cDNA with a commercially available reverse transcription kit. Quantitative real‐time PCR was carried out in 10–20 μL reaction volumes using a SYBR Green/TB Green‐based master mix on a CFX96 real‐time PCR system. Primer sequences are shown in Table S1. Relative mRNA expression was calculated using the 12^−ΔΔCt^ method.

### 2.7 Animals

Male C57BL/6J mice (7 weeks old, 18–20 g) were purchased from Jiangxi Zhonghong Boyuan Biotechnology Co., Ltd. Animals were kept in a specific pathogen‐free facility under controlled temperature and humidity with a 12 h light/12 h dark cycle, with free access to standard chow and water. All experimental procedures adhered to the ARVO *Statement for the Use of Animals in Ophthalmic and Vision Research* and were authorized by the Institutional Animal Care and Use Committee of Nanchang Medical College, and were conducted in accordance with approved ethical guidelines. After a 6 h fasting period, C57BL/6J mice received intraperitoneal injections of streptozotocin (STZ, 50 mg/kg in 0.1 mol/L citrate buffer) once daily for 5 days, whereas control animals were injected with citrate buffer alone. One week after the last STZ injection, mice with tail‐vein blood glucose levels >15 mmol/L were classified as diabetic and randomly assigned to vehicle or chrysin‐treated groups. Chrysin (10 mg/kg) dissolved in 8% dimethyl sulfoxide (DMSO) was administered intraperitoneally once daily for 4 weeks, while diabetic vehicle mice received the same volume of 8% DMSO. Body weight and blood glucose were monitored weekly throughout the study. At the end of treatment, mice were anesthetized with isoflurane and euthanized by cervical dislocation; eyeballs were immediately enucleated, and retinas were dissected for subsequent analyses.

### 2.8 Immunofluorescence and HE staining

Eyes were fixed in 4% paraformaldehyde (PFA) and embedded in paraffin. Serial retinal cross‐sections (5 μm thick) were prepared for both histological and immunofluorescence assays. For histological analysis, sections underwent standard hematoxylin–eosin (H&E) staining and light microscopy. Total retinal thickness (inner limiting membrane to retinal pigment epithelium) was quantified via ImageJ by averaging bilateral measurements taken 200–500 μm from the optic nerve head. For immunofluorescence, sections were permeabilized (0.1% Triton X‐100), blocked (10% goat serum), and sequentially incubated with primary antibodies (4°C, overnight) and fluorescent secondary antibodies, followed by DAPI counterstaining.

### 2.9 Retinal Evans blue assay

Retinal vascular leakage was assessed using Evans blue. Anesthetized mice received a tail‐vein injection of Evans blue (45 mg/kg, 1% in saline; Sigma‐Aldrich, St Louis, MO, USA) and the dye was allowed to circulate for 2 h. Blood was then collected, and mice were perfused via the left ventricle with phosphate‐buffered saline followed by 1% paraformaldehyde. Eyes were enucleated and post‐fixed in 4% paraformaldehyde overnight, after which retinas were dissected and imaged under a stereomicroscope (Leica Microsystems).

### 2.10 RNA sequencing and bioinformatic analysis

BV2 microglial cells were collected for RNA‐seq analysis from the control (CON), high‐glucose vehicle (HG + DMSO), and high‐glucose chrysin‐treated (HG + Chrysin) groups, with four biological replicates per group. Total RNA was isolated with TRIzol reagent according to the manufacturer's instructions, and qualified samples were used for library construction and Illumina sequencing. After sequencing, raw reads were trimmed to remove adapters and low‐quality bases, and clean reads were mapped to the mouse reference genome (mm10) with a splice‐aware aligner. Gene counts were generated and normalized, and differential expression between groups (HG + DMSO vs. CON; HG + Chrysin vs. HG + DMSO) was analyzed using DESeq2, with |log_2_ fold change| > 1 and *p*‐value < 0.05 regarded as significant. Principal component analysis (PCA) was applied to normalized data to compare overall transcriptional profiles. Differentially expressed genes were clustered by hierarchical clustering and displayed as heatmaps to visualize expression patterns across the three conditions. KEGG pathway enrichment and gene set variation analysis (GSVA) were performed in R (version 4.5.1) to identify relevant signaling pathways, with special attention to gene sets containing GBP3 and inflammasome/pyroptosis‐related genes (NLRP3, Il1b, Asc, Casp1, GBP3, and Gsdmd).

### 2.11 Enzyme‐linked immunosorbent assay (ELISA)

Peripheral blood was collected from mice of the NC, DM, DM + DMSO, and DM + Chrysin groups under anesthesia, allowed to clot at room temperature, and centrifuged to obtain serum, which was stored at −80°C until analysis. Serum levels of caspase‐1 and IL‐1β were quantified using commercial mouse ELISA kits (Bioswamp, Wuhan, China) according to the manufacturer's instructions. Briefly, diluted serum samples and standards were added to antibody‐precoated 96‐well plates, incubated, and washed, followed by incubation with HRP‐conjugated detection antibodies and substrate solution. Absorbance was measured at 450 nm with a microplate reader, and cytokine concentrations were calculated from standard curves and expressed as pg/mL.

### 2.12 Statistical analysis

All experiments were independently repeated at least three times, and data are presented as mean ± standard deviation (SD). Statistical analyses were performed using GraphPad Prism version 9.0 (San Diego, CA, USA). Unpaired Student's *t*‐tests and one‐way ANOVA were used for group comparisons. A *p* value < 0.05 was considered statistically significant.

## 3 RESULTS

### 3.1 Effects of chrysin on body weight, blood glucose, retinal structure, and vascular leakage in STZ‐induced diabetic mice

**FIGURE 1:**
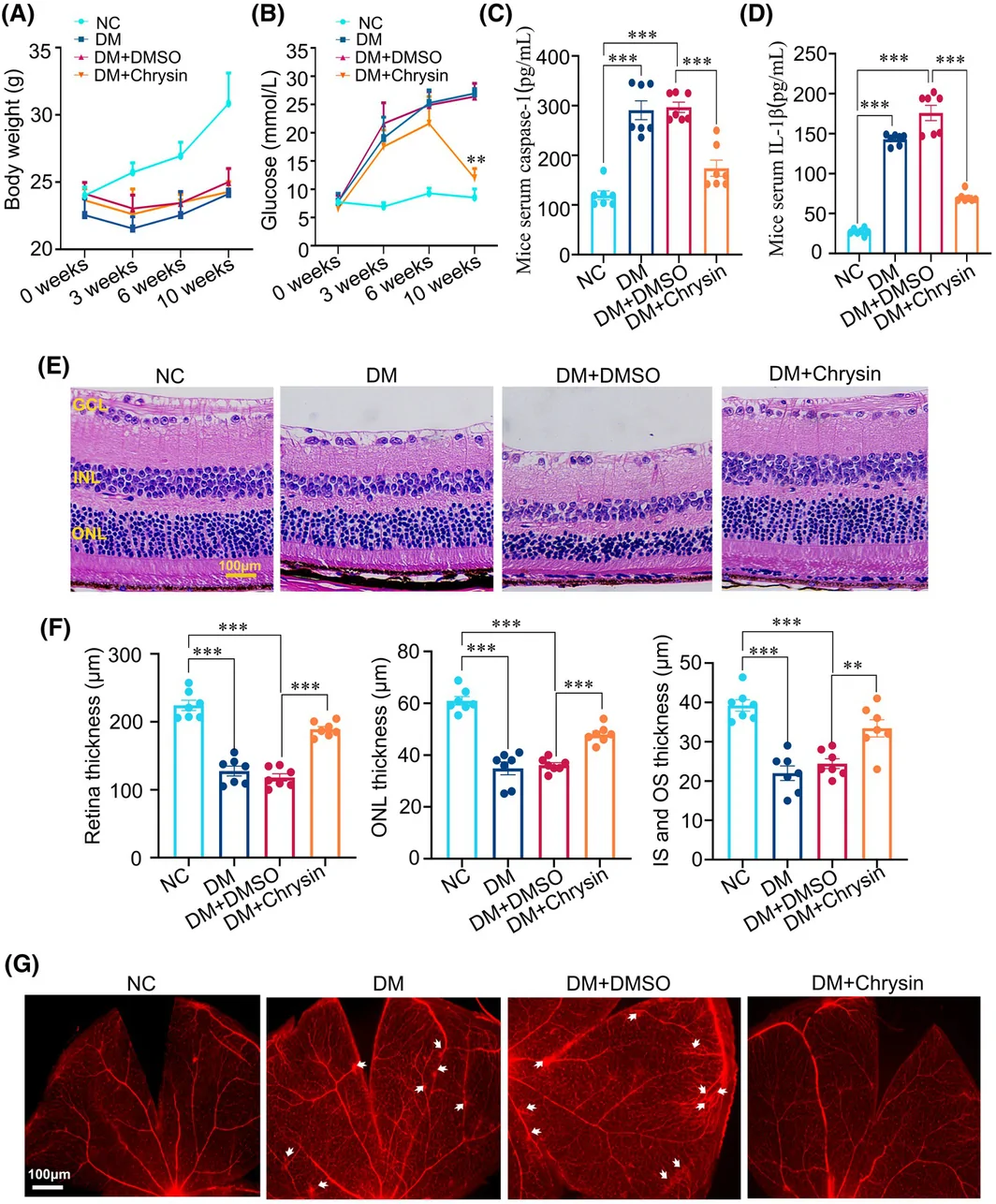
Chrysin mitigates hyperglycemia‐associated inflammation, preserves retinal morphology, and reduces vascular leakage in STZ‐induced diabetic mice. (A) Body weight monitoring in normal control (NC), diabetic (DM), diabetic + vehicle (DM + DMSO), and diabetic + chrysin (DM + Chrysin) groups. (B) Fasting blood glucose levels. (C, D) Serum caspase‐1 (C) and IL‐1β (D) quantified by ELISA. (E) Representative H&E‐stained retinal sections showing neurosensory retinal thinning and layer disorganization in diabetic mice, with partial structural preservation following chrysin treatment. Scale bar = 100 μm. (F) Quantification of total retinal thickness (ganglion cell layer to retinal pigment epithelium), outer nuclear layer (ONL) thickness, and photoreceptor IS/OS thickness (inner/outer segments). (G) Evans blue assay demonstrating retinal vascular leakage (arrows), reduced in chrysin‐treated diabetic mice. Scale bar = 100 μm. Data are mean ± SD (*n* = 7). **p < 0.01, ***p < 0.001, ns = not significant, for indicated comparisons.

After STZ administration, diabetic mice in all groups showed lower body weight than normal controls, and chrysin treatment did not significantly change body weight compared with the diabetic + vehicle group (Figure 1A). Fasting blood glucose levels were markedly higher in diabetic mice than in controls, and were significantly reduced in chrysin‐treated diabetic mice relative to the diabetic + vehicle group (Figure 1B). Serum caspase‐1 and IL‐1β concentrations were increased in diabetic and diabetic + vehicle groups and were decreased in the chrysin‐treated diabetic group (Figure 1C,D).

HE staining showed thinning and disorganization of the neurosensory retina in diabetic mice, with reduction of the outer nuclear layer (ONL) and photoreceptor inner/outer segments. Quantitative measurements confirmed that total retinal thickness (from the ganglion cell layer to the retinal pigment epithelium), ONL thickness, and IS/OS thickness (photoreceptor inner and outer segments) were all decreased in diabetic and diabetic + vehicle mice and were partially preserved in chrysin‐treated diabetic mice (Figure 1E,F). Evans blue assay demonstrated increased retinal vascular leakage in diabetic and diabetic + vehicle mice, whereas leakage was lower in the chrysin‐treated diabetic group (Figure 1G).

### 3.2 Chrysin reduces glial activation, microglial accumulation, and retinal cell apoptosis in diabetic mice

**FIGURE 2:**
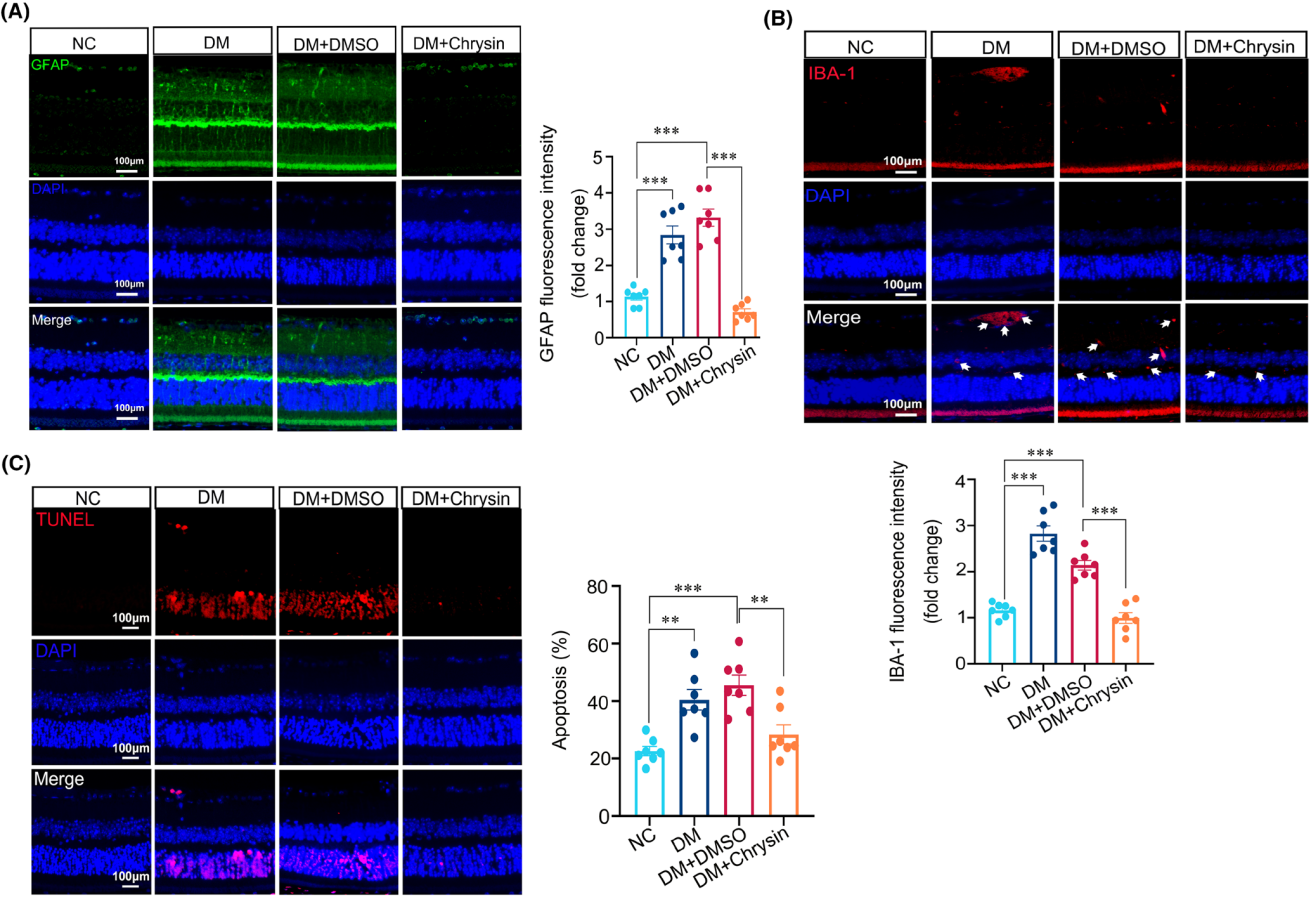
Chrysin attenuates retinal glial activation and apoptosis in STZ‐induced diabetic mice. (A) GFAP immunofluorescence (DAPI, nuclear staining) and quantification of GFAP fluorescence intensity in NC, DM, DM + DMSO, and DM + Chrysin groups. (B) IBA‐1 immunofluorescence showing increased IBA‐1‐positive microglia/macrophages in diabetic retinas (arrows) and reduced signals after chrysin treatment, with quantification of IBA‐1 fluorescence intensity. (C) TUNEL staining (DAPI, nuclear staining) and quantification of apoptotic cell ratio. Scale bar = 100 μm. Data are mean ± SD (*n* = 7). **p < 0.01,***p < 0.001, ns = not significant, for indicated comparisons.

Immunofluorescence staining for GFAP (expressed by Müller glial cells and astrocytes and markedly upregulated after injury and in disease states^8^) showed weak labeling largely restricted to the nerve fiber–ganglion cell layer in control retinas, whereas diabetic mice displayed robust GFAP upregulation extending across the inner retina; a similar pattern was observed in the diabetic + vehicle mice. Chrysin‐treated diabetic mice exhibited visibly reduced GFAP staining, and quantitative analysis confirmed a significant decrease in GFAP fluorescence intensity compared with the diabetic and diabetic + vehicle groups (Figure 2A). Staining for IBA‐1 (a characteristic marker expressed by retinal microglia/macrophages) revealed sparse, weakly labeled microglia/macrophages in control retinas, while diabetic and diabetic + vehicle mice showed increased IBA‐1‐positive signals and apparent redistribution of microglia/macrophages within the retinal layers. In chrysin‐treated diabetic mice, IBA‐1 fluorescence intensity was significantly lower than in diabetic and diabetic + vehicle groups (Figure 2B). In line with this, TUNEL staining demonstrated few apoptotic nuclei in control retinas but a marked increase in diabetic and diabetic + vehicle mice, whereas chrysin treatment reduced the proportion of TUNEL‐positive cells (Figure 2C).

### 3.3 RNA‐seq reveals chrysin‐responsive pyroptosis signatures in high‐glucose–stimulated BV2 microglia

**FIGURE 3:**
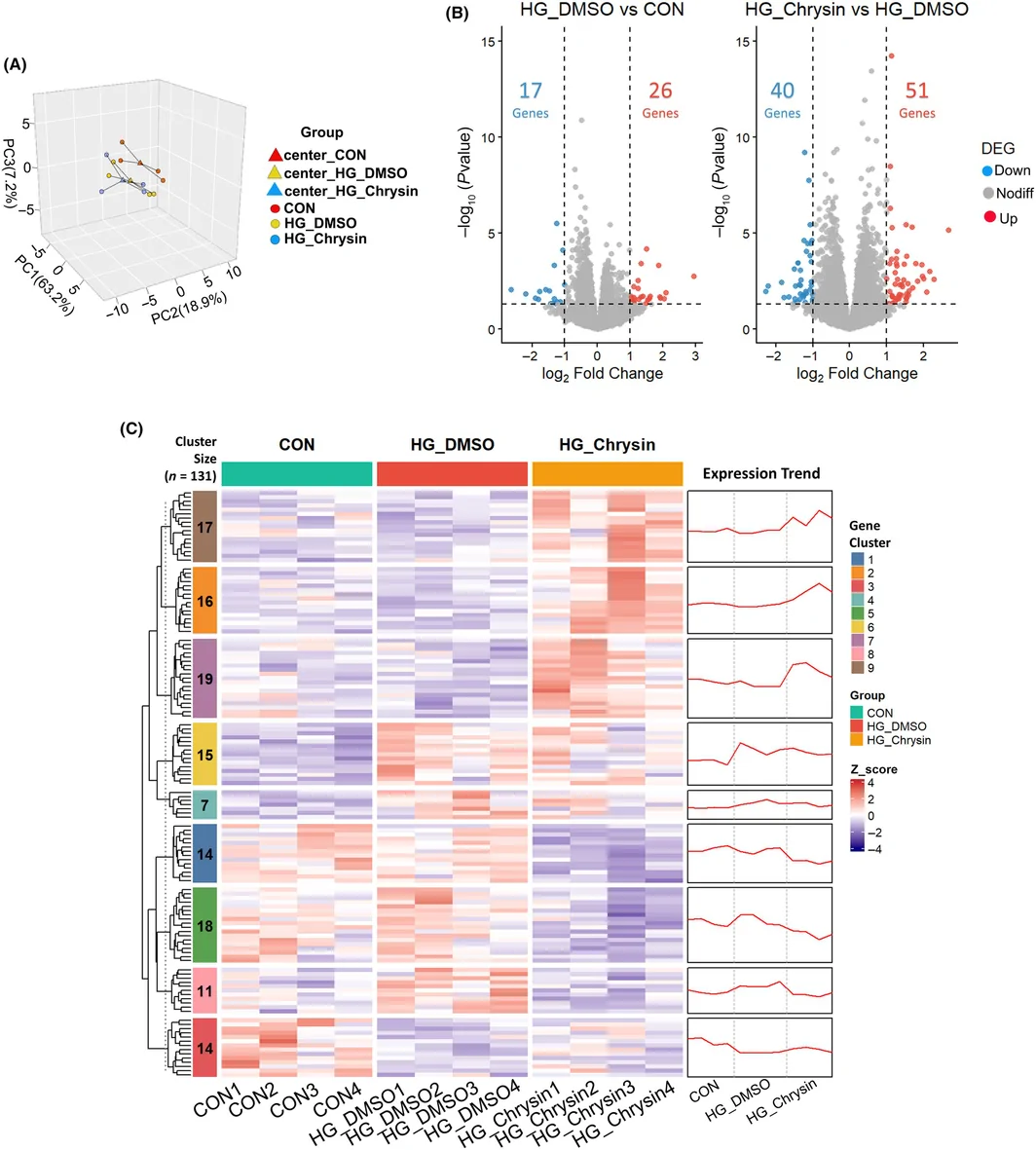
RNA‐seq profiling of BV2 microglia under high glucose and chrysin treatment. (A) Principal component analysis (PCA) of RNA‐seq data from BV2 cells in control (CON), high‐glucose vehicle (HG_DMSO), and high‐glucose chrysin‐treated (HG_Chrysin) groups (*n* = 4 biological replicates per group). (B) Volcano plots showing differentially expressed genes (DEGs) for HG_DMSO versus CON and HG_Chrysin versus HG_DMSO. (C) Hierarchical clustering heatmap of DEGs showing expression modules across conditions.

To characterize transcriptomic changes induced by high glucose and their modulation by chrysin, RNA‐seq was performed in BV2 cells from the control (CON), high‐glucose vehicle (HG_DMSO), and high‐glucose chrysin‐treated (HG_Chrysin) groups. Principal component analysis (PCA) showed clear separation among the three conditions, indicating distinct global expression profiles (Figure 3A). Differential expression analysis identified 43 DEGs in HG_DMSO versus CON (26 upregulated and 17 downregulated) (Figure 3B). Comparison of HG_Chrysin versus HG_DMSO revealed a larger transcriptional shift (91 DEGs; 51 upregulated and 40 downregulated), suggesting broad gene regulation following chrysin treatment under high‐glucose conditions (Figure 3B). Hierarchical clustering of DEGs further resolved multiple expression modules with distinct trends across groups, with several clusters showing high‐glucose‐induced changes that were partially reversed in HG_Chrysin (Figure 3C).

**FIGURE 4:**
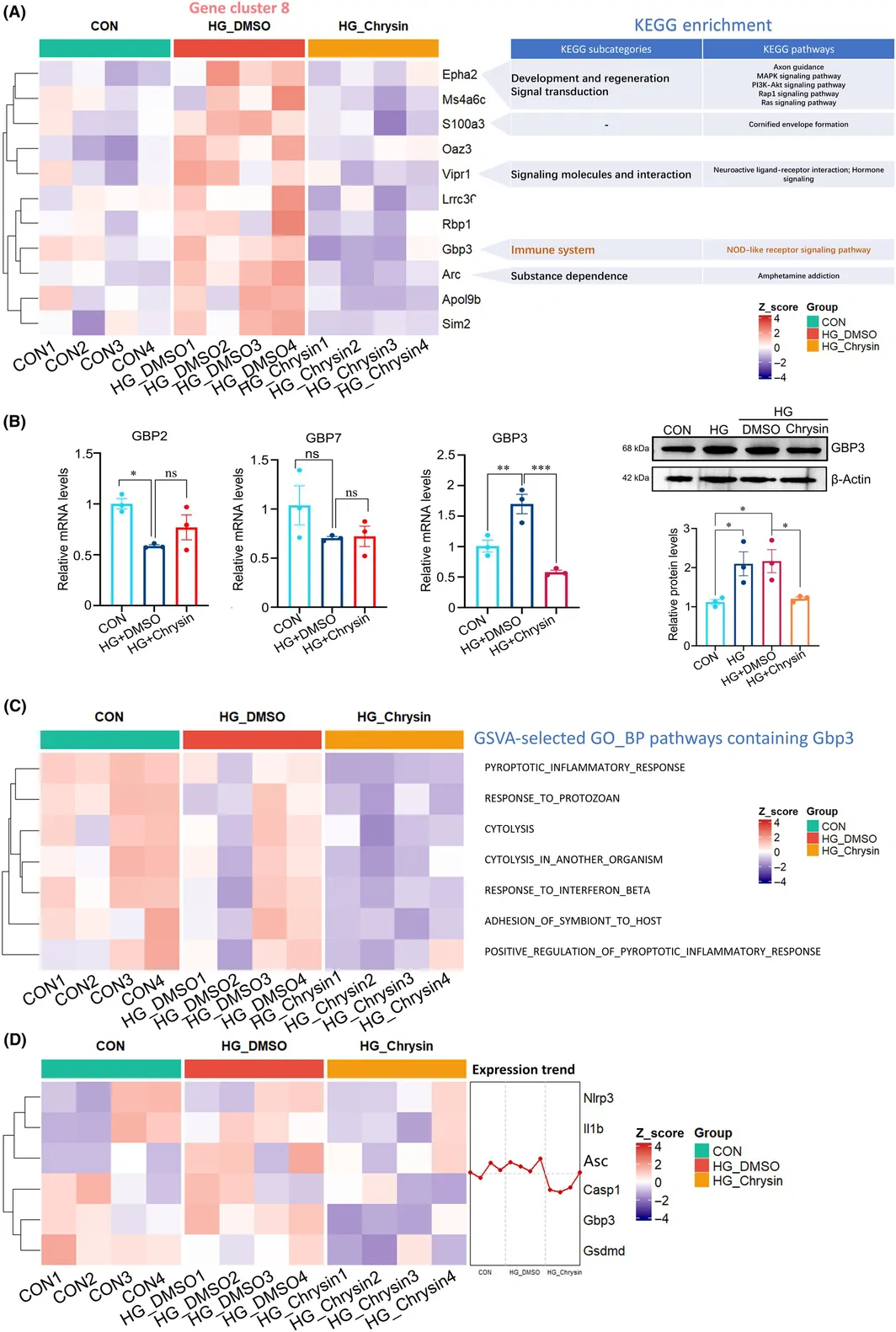
GBP3‐linked pyroptosis signature is reduced by chrysin in BV2 microglia. (A) Heatmap of DEG Cluster 8 with KEGG enrichment, highlighting immune‐related pathways including NOD‐like receptor signaling. (B) RT‐qPCR validation of Gbp2, GBP3, and Gbp7, and Western blot validation of GBP3 protein with quantification. (C) GSVA of GO biological process terms containing GBP3 across groups. (D) Heatmap of inflammasome/pyroptosis‐related genes (NLRP3, Il1b, Asc, Casp1, GBP3, and Gsdmd) with expression trend plot. Data are mean ± SD (*n* = 3–4); *p < 0.05, **p < 0.01, ***p < 0.001, ns = not significant, for indicated comparisons.

Among these modules, a representative gene cluster (Cluster 8) displayed prominent induction in HG_DMSO and attenuation in HG_Chrysin (Figure 4A). KEGG enrichment of this cluster highlighted pathways related to signal transduction and immune‐associated signaling, including the NOD‐like receptor signaling pathway (Figure 4A). Given the presence of GBP3 within this cluster, we further investigated guanylate‐binding protein genes: RT‐qPCR showed that GBP3 was significantly upregulated in HG_DMSO and reduced by chrysin, whereas Gbp2 and Gbp7 exhibited comparatively modest or non‐significant changes (Figure 4B). Consistent with this finding, Western blot confirmed increased GBP3 protein under high glucose/vehicle and decreased GBP3 following chrysin treatment (Figure 4B, supplementary file). In line with these findings, GSVA of GO biological process terms containing GBP3 demonstrated higher scores in HG_DMSO and lower scores in HG_Chrysin for several inflammation/immune‐related programs, including pyroptosis‐associated signatures (Figure 4C). Moreover, a focused heatmap indicated coordinated regulation of inflammasome/pyroptosis‐related genes (NLRP3, Il1b, Asc, Casp1, and Gsdmd) with an overall pattern of elevation in HG_DMSO and reduction in HG_Chrysin (Figure 4D).

### 3.4 Chrysin alleviates high‐glucose‐induced oxidative stress and pyroptosis‐associated responses in BV2 microglia

**FIGURE 5:**
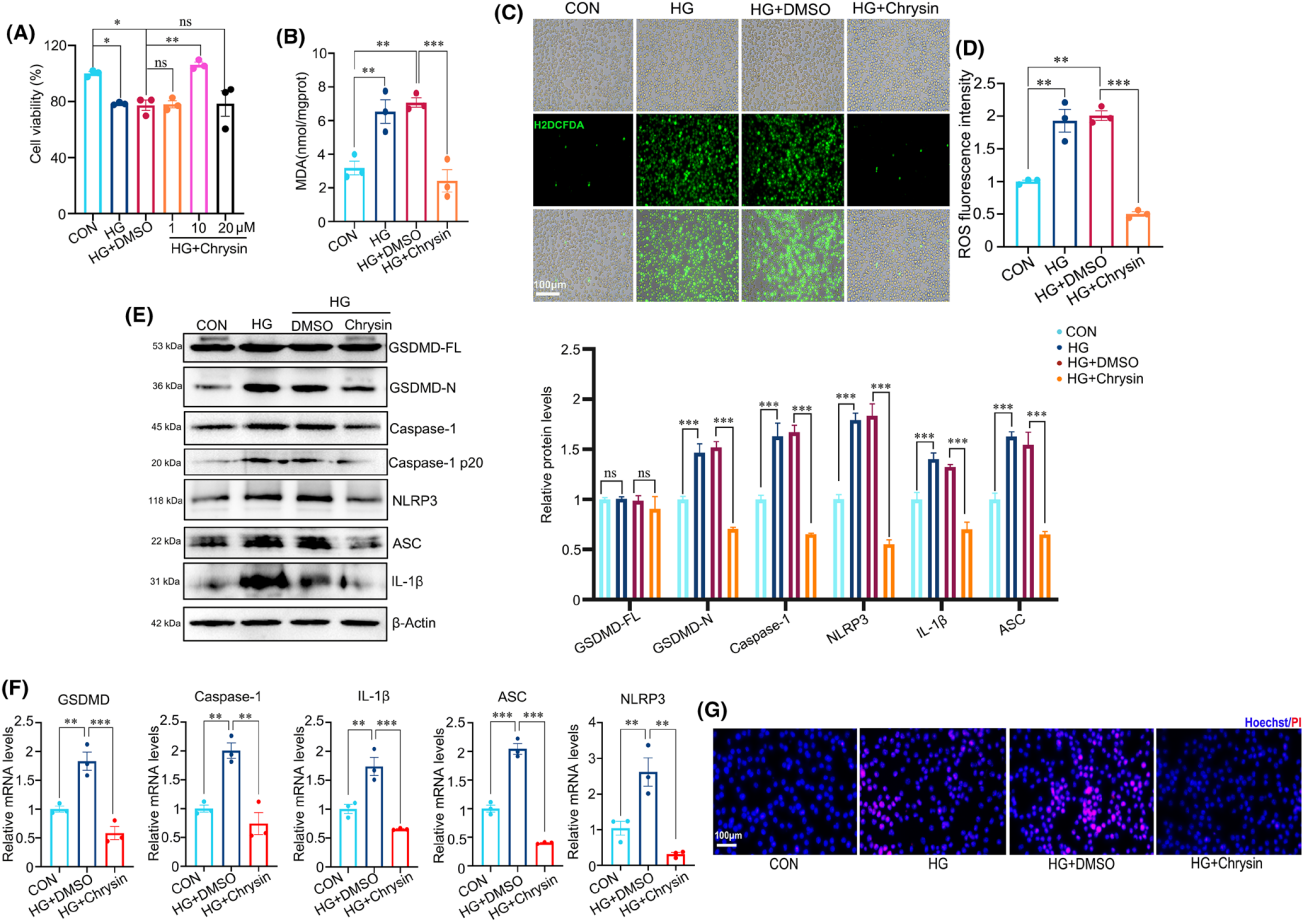
Chrysin attenuates oxidative stress and pyroptosis‐related responses in HG‐stimulated BV2 microglia. (A) CCK‐8 assay of cell viability after HG exposure with vehicle or graded doses of chrysin. (B) MDA content. (C) Representative H2DCFDA images of intracellular ROS. Scale bar = 100 μm. (D) Quantification of ROS fluorescence intensity. (E) Western blot of GSDMD, GSDMD‐N, caspase‐1, caspase‐1 p20, NLRP3, ASC, and IL‐1β (β‐Actin as loading control). (F) RT‐qPCR of Gsdmd, Casp1, Il1b, Asc, and NLRP3. (G) Hoechst/PI staining showing PI‐positive cells. Scale bar = 100 μm. Data are mean ± SD (*n* = 3); *p < 0.05, **p < 0.01, ***p < 0.001, ns = not significant, for indicated comparisons.

To evaluate the effects of chrysin in vitro, BV2 cells were exposed to high glucose (HG) with vehicle or chrysin treatment. CCK‐8 assays showed that HG reduced BV2 viability compared with control, whereas chrysin improved cell viability in a concentration‐dependent manner, with 10 μmol/L producing the most evident recovery and therefore being used for subsequent experiments (Figure 5A). In agreement, HG increased lipid peroxidation, as reflected by elevated MDA levels, while chrysin significantly decreased MDA under HG conditions (Figure 5B). HG also markedly enhanced intracellular ROS production, demonstrated by increased H2DCFDA fluorescence and quantified ROS intensity, both of which were reduced by chrysin (Figure 5C,D).

**FIGURE 6:**
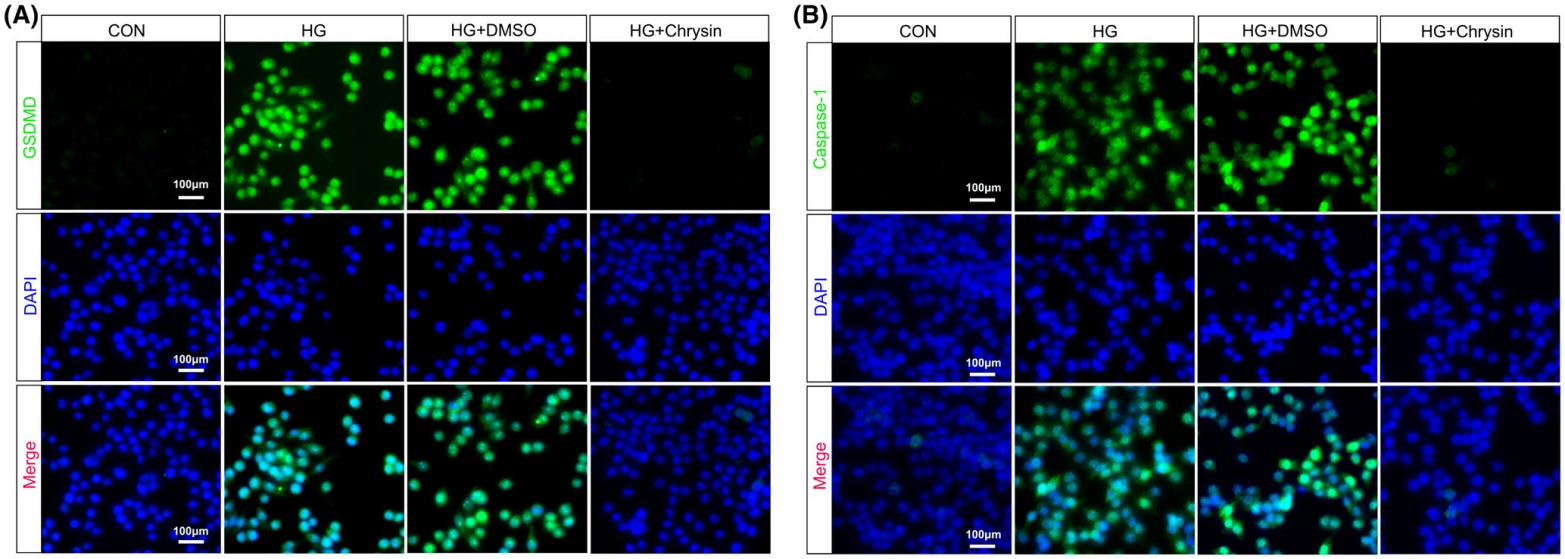
Chrysin reduces HG‐induced GSDMD and caspase‐1 immunoreactivity in BV2 microglia. Representative immunofluorescence images of GSDMD (A) and caspase‐1 (B) with DAPI nuclear staining in CON, HG, HG + DMSO, and HG + Chrysin groups. Scale bar = 100 μm.

Given the RNA‐seq indication of pyroptosis‐related signatures, we examined inflammasome/pyroptosis‐associated molecules. Western blot revealed that HG increased the levels of NLRP3, ASC, caspase‐1, cleaved caspase‐1 (p20), GSDMD‐FL, IL‐1β, and cleaved GSDMD (GSDMD‐N), whereas chrysin treatment reduced these protein signals (Figure 5E, supplementary file). RT‐qPCR further showed that HG upregulated NLRP3, Asc, Casp1, Il1b, and Gsdmd mRNA expression, which was significantly downregulated by chrysin (Figure 5F). In parallel, Hoechst/PI staining demonstrated more PI‐positive cells under HG and HG + vehicle conditions, while chrysin decreased PI positivity (Figure 5G). Immunofluorescence staining corroborated these findings at the cellular level: HG increased GSDMD and caspase‐1 fluorescence signals, which were markedly weakened in the HG + Chrysin group (Figure 6A,B).

### 3.5 GBP3 knockdown suppresses HG‐induced pyroptosis‐associated signaling in BV2 microglia

**FIGURE 7:**
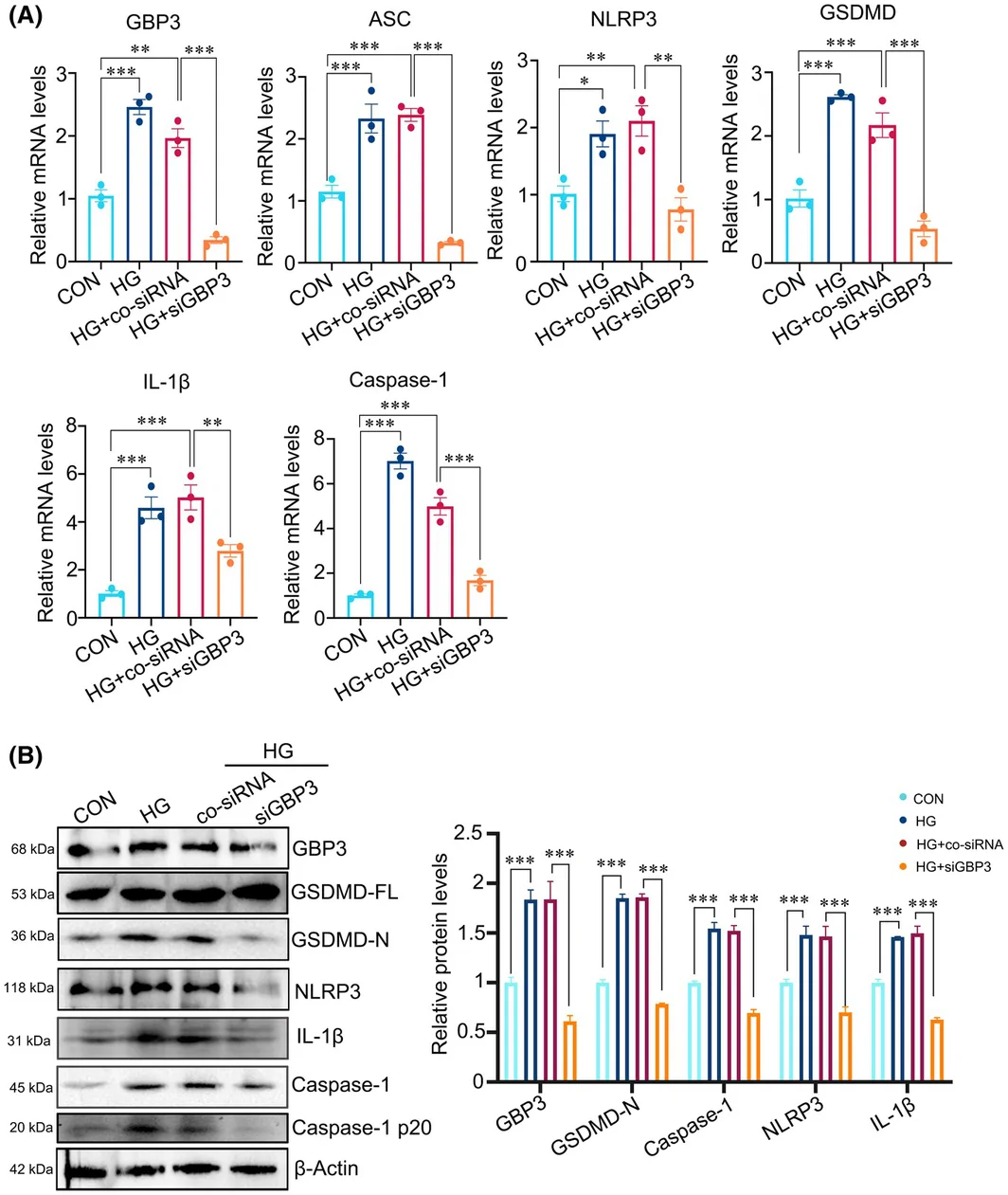
GBP3 knockdown attenuates HG‐associated activation of the NLRP3–GSDMD axis in BV2 microglia. (A) RT‐qPCR analysis of GBP3 and pyroptosis/inflammasome‐related transcripts (Asc, NLRP3, Gsdmd, Casp1, and Il1b) in CON, HG, HG with control siRNA (HG + co‐siRNA), and HG with GBP3 siRNA (HG + siGBP3) groups. (B) Representative Western blots and densitometric quantification of GBP3, NLRP3, GSDMD and cleaved GSDMD (GSDMD‐N), as well as IL‐1β and caspase‐1 p20, with β‐Actin as the loading control. Data are mean ± SD (*n* = 3); *p < 0.05, **p < 0.01, ***p < 0.001, ns = not significant, for indicated comparisons.

To interrogate the role of GBP3 in HG‐triggered pyroptosis‐related responses, BV2 cells were transfected with control siRNA (co‐siRNA) or GBP3 siRNA (siGBP3) and then exposed to HG. RT‐qPCR confirmed effective GBP3 silencing under HG conditions (Figure 7A). Compared with the HG and HG + co‐siRNA groups, siGBP3 significantly reduced the HG‐induced upregulation of Asc, NLRP3, Gsdmd, Casp1 and Il1b transcripts (Figure 7A). Consistent with this finding, Western blotting showed that HG increased GBP3 protein levels, which was accompanied by elevated NLRP3, IL‐1β, caspase‐1, cleaved caspase‐1 (p20) and cleaved GSDMD (GSDMD‐N); these protein changes were markedly attenuated by GBP3 knockdown (Figure 7B, supplementary file). Collectively, these results indicate that GBP3 contributes to HG‐associated activation of the NLRP3–GSDMD axis in BV2 cells.

### 3.6 Chrysin suppresses NLRP3 inflammasome/pyroptosis‐associated signaling in retinas of diabetic mice

**FIGURE 8:**
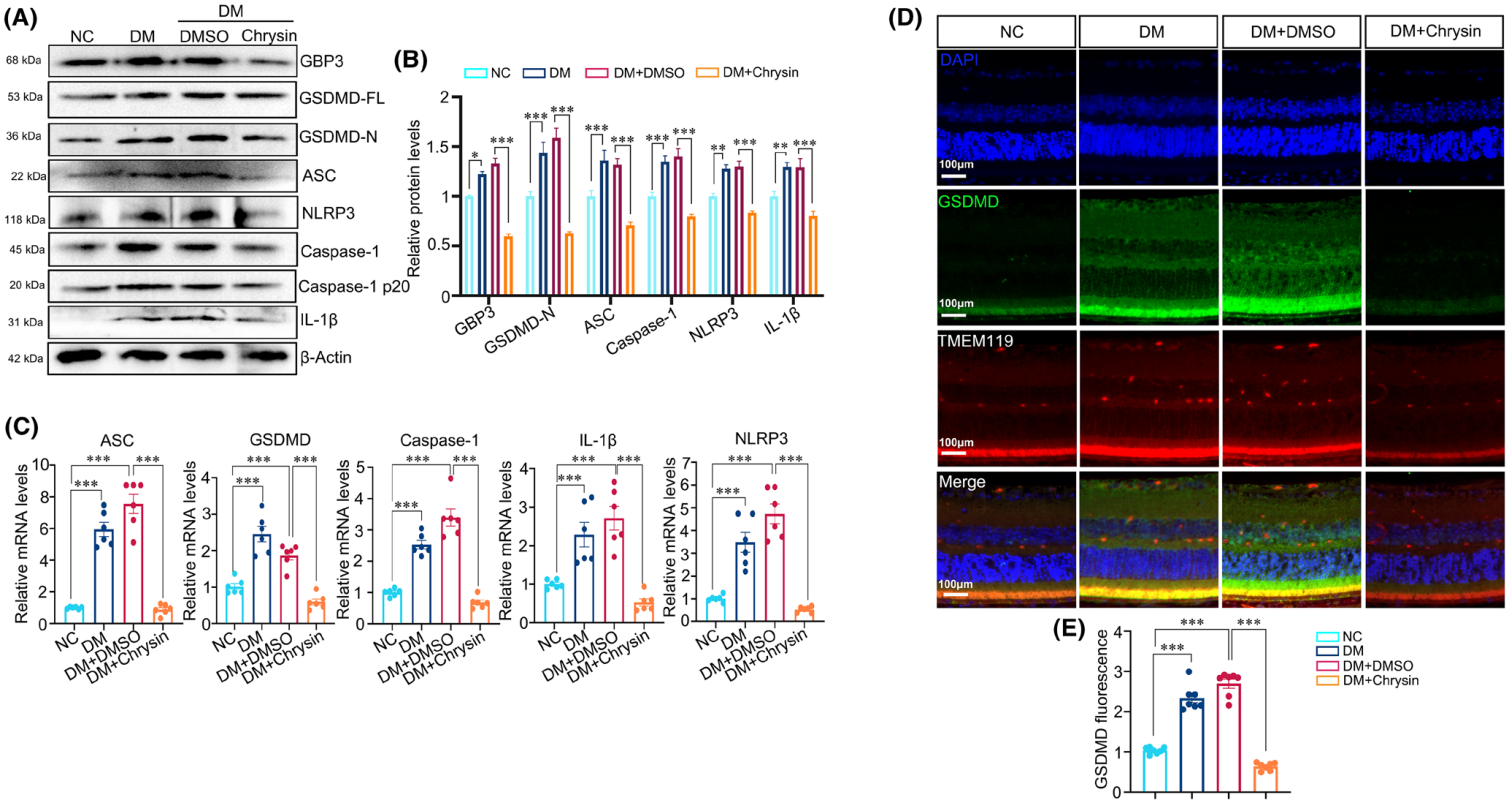
Chrysin suppresses retinal inflammasome/pyroptosis signaling in diabetic mice. (A) Representative Western blot of GBP3, cleaved GSDMD (GSDMD‐N), GSDMD, ASC, NLRP3, caspase‐1, caspase‐1 p20 and IL‐1β in retinas from normal control (NC), diabetic (DM), diabetic treated with vehicle (DM + DMSO), and diabetic treated with chrysin (DM + Chrysin) groups, with β‐Actin as the loading control. (B) Densitometric quantification of the corresponding protein levels. (C) RT‐qPCR analysis of Asc, Gsdmd, Casp1, Il1b, and NLRP3 mRNA expression in retinal tissues across groups. (D) Representative retinal immunofluorescence images of GSDMD and TMEM119 co‐staining with DAPI nuclear staining. Scale bar = 100 μm. (E) Quantification of GSDMD fluorescence intensity. Data are mean ± SD (*n* = 3–7); *p < 0.05, **p < 0.01, ***p < 0.001, ns = not significant, for indicated comparisons.

To examine inflammasome‐ and pyroptosis‐related signaling in vivo, retinal tissues from normal control and diabetic mice treated with vehicle or chrysin were analyzed. Western blot showed increased levels of GBP3, cleaved GSDMD (GSDMD‐N), ASC, NLRP3, caspase‐1, cleaved caspase‐1 (p20) and IL‐1β in retinas from diabetic mice and vehicle‐treated diabetic mice compared with controls, whereas these protein signals were markedly reduced in chrysin‐treated diabetic mice (Figure 8A,B, supplementary file). In addition, RT‐qPCR demonstrated that diabetic retinas exhibited significant upregulation of Asc, Gsdmd, Casp1, Il1b, and NLRP3 transcripts, and chrysin treatment significantly decreased the expression of these genes (Figure 8C). Double immunofluorescence staining for GSDMD and the microglia‐specific marker TMEM119 further confirmed that the enhanced GSDMD signals primarily co‐localized with microglia. Specifically, diabetic and vehicle‐treated diabetic retinas exhibited robust GSDMD expression within TMEM119‐positive cells, while chrysin‐treated diabetic mice showed markedly weaker GSDMD fluorescence in these cells. Quantitative analysis supported a significant reduction in overall GSDMD fluorescence intensity following chrysin treatment (Figure 8D,E).

## 4 DISCUSSION

Growing evidence across diverse disease settings supports pyroptosis—a lytic, pro‐inflammatory form of regulated cell death driven by inflammasome–caspase activation and gasdermin pore formation—as a druggable pathogenic axis, making its key nodes (inflammasome sensors, inflammatory caspases, and gasdermins) attractive targets for therapeutic intervention.^13^, ^14^, ^15^ In parallel with synthetic inhibitors under active development, natural bioactive compounds have received increasing attention as multi‐target modulators capable of dampening pyroptosis‐related signaling, often by restraining NLRP3 inflammasome activation and/or limiting downstream gasdermin‐mediated membrane permeabilization.^16^, ^17^, ^18^

Chrysin (5,7‐dihydroxyflavone) is a naturally occurring flavone found in propolis/honey and various medicinal plants, and is widely reported to exert antioxidant and anti‐inflammatory bioactivities with neuroprotective potential.^19^, ^20^ Notably, independent studies indicate that chrysin can restrain NLRP3 inflammasome‐linked inflammatory signaling in different experimental contexts.^21^, ^22^ In our work, chrysin treatment in STZ‐induced diabetic mice reduced fasting glucose, lowered circulating caspase‐1 and IL‐1β, partially preserved total retinal thickness, and decreased Evans blue‐measured vascular leakage. In the retina, chrysin was associated with reduced GFAP and IBA‐1 signals and fewer TUNEL‐positive cells. In high‐glucose‐stimulated BV2 microglia, RNA‐seq identified chrysin‐responsive pyroptosis‐related signatures, including a GBP3‐containing module, and subsequent validation showed that chrysin decreased oxidative stress (ROS and MDA), improved cell viability, and reduced NLRP3/ASC/caspase‐1/IL‐1β and GSDMD cleavage at transcript and protein levels. Mechanistically, silencing GBP3 reduced HG‐induced activation of the NLRP3–GSDMD pathway in BV2 cells, and retinas from chrysin‐treated diabetic mice showed concordant decreases in inflammasome/pyroptosis markers, including reduced GSDMD immunofluorescence.

Oxidative stress is a central pathogenic stressor in diabetic retinopathy, with sustained ROS accumulation amplifying inflammatory signaling and damaging multiple components of the retinal neurovascular unit, including glia.^23^, ^24^ Müller glia are increasingly recognized as key contributors to this redox–inflammatory milieu in diabetes, and activated microglia can further propagate oxidative and cytokine‐driven injury.^25^, ^26^ In parallel, pyroptosis has emerged as an important inflammatory cell‐death program in diabetic retinal disease: multiple studies and reviews implicate the NLRP3 inflammasome–caspase‐1–GSDMD axis in retinal neurovascular unit dysfunction, with high glucose capable of triggering NLRP3/caspase‐1/GSDMD‐dependent pyroptosis in retinal cells.^27^, ^28^, ^29^, ^30^, ^31^ A key challenge in deciphering retinal pyroptosis is its multi‐cellular nature, as non‐microglial cells—including Müller glia, endothelial cells, and pericytes—can also express pyroptotic machinery under diabetic stress.^32^ To definitively address whether microglial pyroptosis occurs in vivo and is directly targeted by chrysin, we performed dual immunofluorescence co‐localization staining of GSDMD with TMEM119, a highly specific marker for resident retinal microglia, on retinal tissue sections.^33^ Our results demonstrated a marked induction of GSDMD expression within TMEM119‐positive resident microglia in the diabetic retina. Importantly, treatment with chrysin substantially attenuated GSDMD immunoreactivity specifically within these TMEM119‐positive cells. Also, our cellular model show that chrysin markedly reduced oxidative stress in HG‐stimulated BV2 microglia and concomitantly suppressed pyroptosis‐associated readouts, with consistent inhibition of inflammasome/pyroptosis markers also observed in diabetic retinas following chrysin treatment. This direct cellular intersection confirms that resident retinal microglia undergo GSDMD‐mediated pyroptosis under hyperglycemic conditions, and confirms that chrysin directly mitigates microglial pyroptosis in vivo, connecting our animal model observations with cell‐based mechanisms. These observations are mechanistically consistent with current DR concepts, in which sustained oxidative stress and NLRP3 inflammasome activation contribute to retinal injury; thus, chrysin's documented antioxidant and anti‐inflammatory activities, together with its reported capacity to restrain NLRP3 signaling, provide a plausible basis for its effects in diabetic retinal disease.

Interferon‐inducible guanylate‐binding proteins (GBPs) have emerged as important upstream regulators of inflammasome‐driven pyroptosis, functioning at the interface of pathogen/danger sensing and downstream execution pathways. GBPs can facilitate noncanonical inflammasome activation (caspase‐11/4/5 in rodents/humans) and promote the subsequent engagement of canonical inflammasomes such as NLRP3, thereby amplifying caspase‐1 activation and inflammatory cytokine maturation.^34^, ^35^, ^36^, ^37^, ^38^ Within this family, GBP3 has been increasingly linked to the cell's pyroptotic machinery: a synthesis of mechanistic studies in the field indicates that GBP3 can participate in gasdermin D (GSDMD) pore‐forming execution by facilitating GSDMD‐NT assembly/trafficking after cleavage, providing a plausible route by which GBP3 may tune pyroptosis intensity.^39^ Consistent with this concept, recent disease‐model studies report that GBP3 silencing reduces NLRP3 and GSDMD (and related pyroptosis markers), supporting GBP3 as a regulator of the NLRP3–GSDMD axis.^40^ More broadly, multiple infection models demonstrate that chromosome 3‐encoded GBPs (including GBP3) contribute to inflammasome‐dependent cell death and couple caspase‐11 activity to NLRP3 signaling, reinforcing a conserved functional link between GBPs and inflammasome/pyroptosis outputs.^35^, ^36^, ^37^, ^38^, ^41^ In line with this literature, our BV2 data showing that GBP3 knockdown decreases HG‐associated NLRP3 and Gsdmd expression and reduces GSDMD cleavage support a model in which GBP3 acts upstream of key pyroptosis effectors in microglia.

While our findings provide strong evidence for a chrysin‐regulated GBP3–NLRP3–GSDMD axis in microglial pyroptosis, several limitations need to consideration. Although GSDMD/TMEM119 co‐staining confirms microglial pyroptosis, future studies utilizing multi‐color co‐localization with endothelial (e.g., CD31) or Müller glial (e.g., GFAP) markers will further elucidate whether chrysin exerts complementary anti‐pyroptotic actions on non‐immune retinal neurovascular unit components. Furthermore, we established mechanistic knockdowns in immortalized BV2 microglia; however, although widely adopted for neuroinflammation research, BV2 cells cannot fully recapitulate the phenotype of primary retinal microglia residing in the native retinal microenvironment. Confirming these signaling dynamics in primary retinal microglia and employing microglia‐specific GBP3 conditional knockout mice alongside electroretinography (ERG) will provide definitive genetic and functional proof. Finally, retinal exposure and pharmacokinetic parameters of chrysin were not quantified in this study, making the development of targeted ocular delivery formulations an important next step to enhance retinal bioavailability and clinical translation.

Overall, our findings support a microglia‐focused mechanism by which chrysin modulates diabetic retinal disease, linking in vivo observations in STZ‐diabetic mice with BV2 transcriptomic profiling and targeted functional assays. Notably, using RNA‐seq and siRNA‐mediated knockdown, we identify GBP3 as a chrysin‐responsive regulator that connects high‐glucose stimulation to activation of the NLRP3–GSDMD pyroptosis pathway in microglia. Collectively, these findings provide a solid theoretical basis for the clinical application of chrysin in the treatment of diabetic retinopathy.

## 5 CONCLUSIONS

This study demonstrates that the natural flavonoid chrysin ameliorates key pathological features of experimental diabetic retinopathy. Beyond its systemic metabolic and anti‐inflammatory effects, chrysin confers retinal protection that is accompanied by reduced glial activation, diminished vascular leakage, and decreased retinal cell apoptosis. Mechanistically, our data indicate that suppression of oxidative stress and NLRP3 inflammasome‐driven pyroptosis represents a central mode of chrysin's action within the retinal neurovascular unit, with a particular emphasis on microglia. Integrating RNA‐seq with functional validation, we identified GBP3 as a previously unrecognized chrysin‐responsive target and an upstream regulator of the NLRP3–GSDMD axis in high‐glucose‐stimulated microglia. Collectively, these findings suggest chrysin as a multi‐target therapeutic candidate for concurrently addressing oxidative injury, neuroinflammation, and pyroptosis‐related mechanisms in diabetic retinal disease, and indicate that GBP3 is a potential glia‐oriented target for innovative interventions in diabetic retinopathy and related neurovascular disorders.

## AUTHOR CONTRIBUTIONS

**Qun Liu:** Writing – original draft; methodology; funding acquisition; investigation; formal analysis; project administration. **Song‐min Wang:** Investigation; validation; data curation; methodology. **Xuan Chen:** Validation; methodology; investigation. **Yi‐rao You:** Investigation; methodology; validation; data curation. **Chen‐jiao Guo:** Validation; methodology; investigation. **Chang‐qin Xie:** Methodology; conceptualization; investigation. **Lu‐lu Wang:** Methodology; investigation. **Qi Liu:** Writing – review and editing. **Lan Zhang:** Project administration; software. **Jun‐tao Yang:** Writing – review and editing; conceptualization; funding acquisition; formal analysis; project administration.

## FUNDING INFORMATION

This study was financially supported by the Jiangxi Provincial Natural Science Foundation (20242BAB20389), the Early‐Career Young Scientists and Technologists Project of Jiangxi Province (20244BCE52257), the Doctoral Research Initiation Fund Project of Nanchang Medical College (NYB24004), and the Research and Innovation Team of Nanchang Medical College (NYTD202409, NYTD202408).

## CONFLICT OF INTEREST STATEMENT

The authors declare no known competing financial interests or personal relationships that could have influenced the work reported in this paper.

## ETHICS STATEMENT

The Animal Welfare and Ethics Review Form has been uploaded to the attachments, with the assigned number NYLLSC20260003.

## Supporting information


**Data S1:** Supplementary File.

**Supplementary Table 1.** Primers used for quantitative RT‐PCR.

## Data Availability

The data that supports the findings of this study are available in the Supporting Information of this article.
