# Supplementary material for: Chrysin attenuates diabetes‐induced retinal injury by inhibiting microglial GBP3/NLRP3/GSDMD‐mediated pyroptosis
Source: Animal Model Exp Med. 2026 Sep 24;9(10):2030–43. doi: 10.1002/ame2.70293 (PMC13602009; doi:10.1002/ame2.70293)
Supplement: Supplementary file 1 [file AME2-9-2030-s001.pdf]

Fig4. B

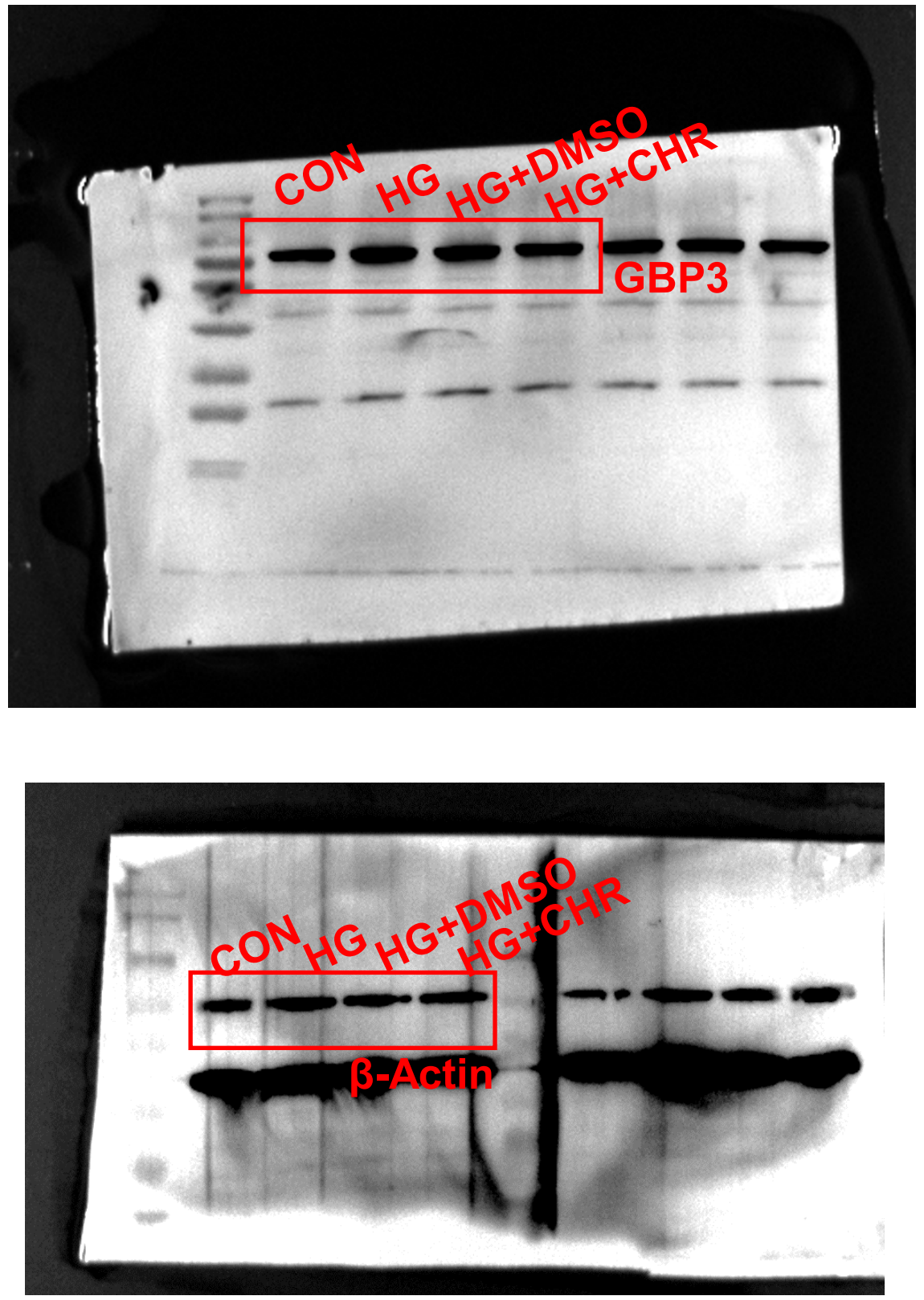
CON
HG
HG+DMSO
HG+CHR
GBP3
CON
HG
HG+DMSO
HG+CHR
β-Actin

Fig5. C

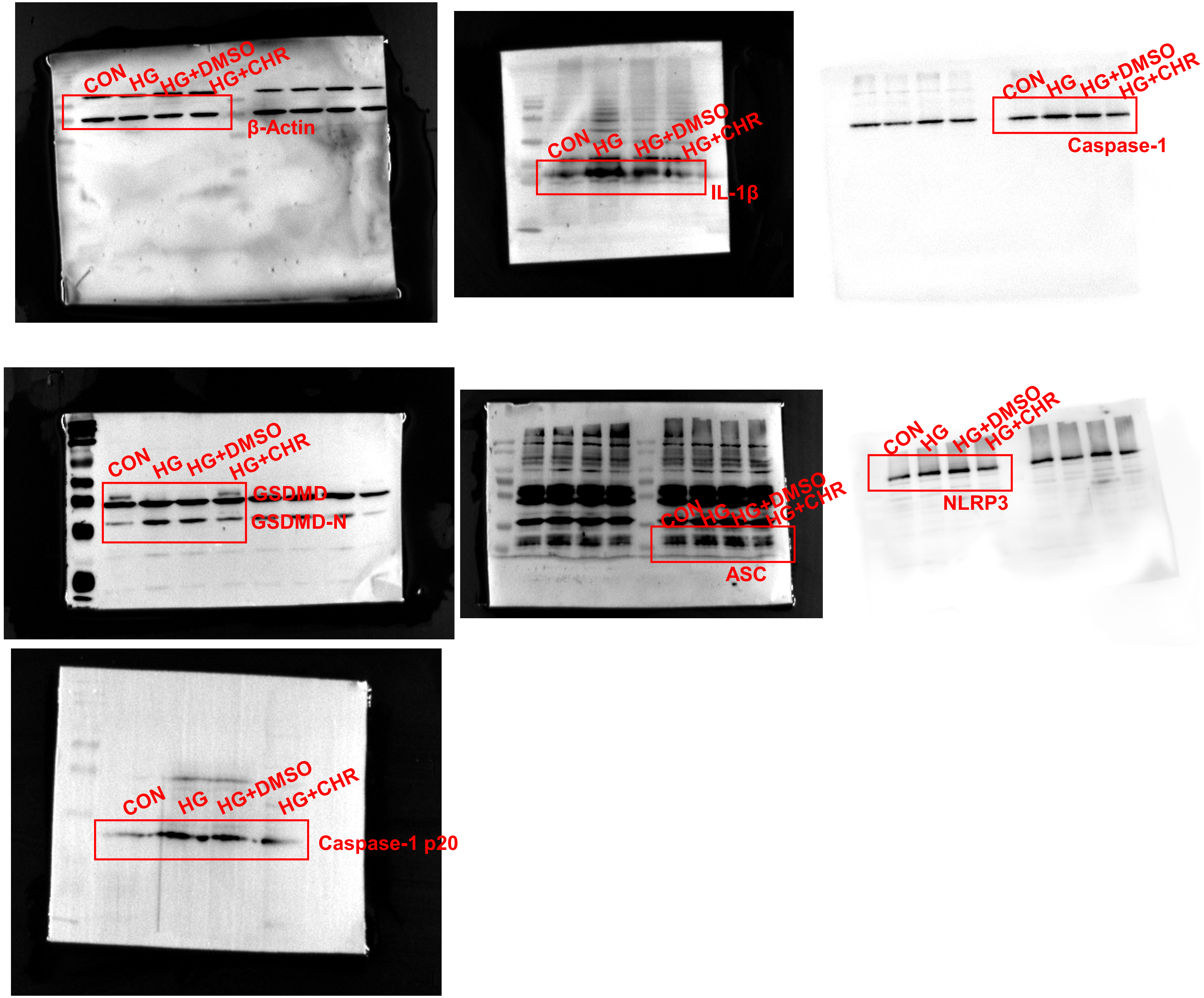
CON
HG
HG+DMSO
HG+CHR
β-Actin
CON
HG
HG+DMSO
HG+CHR
IL-1β
CON
HG
HG+DMSO
HG+CHR
Caspase-1
CON
HG
HG+DMSO
HG+CHR
GSDMD
GSDMD-N
CON
HG
HG+DMSO
HG+CHR
ASC
CON
HG
HG+DMSO
HG+CHR
NLRP3
CON
HG
HG+DMSO
HG+CHR
Caspase-1 p20

Fig7. B

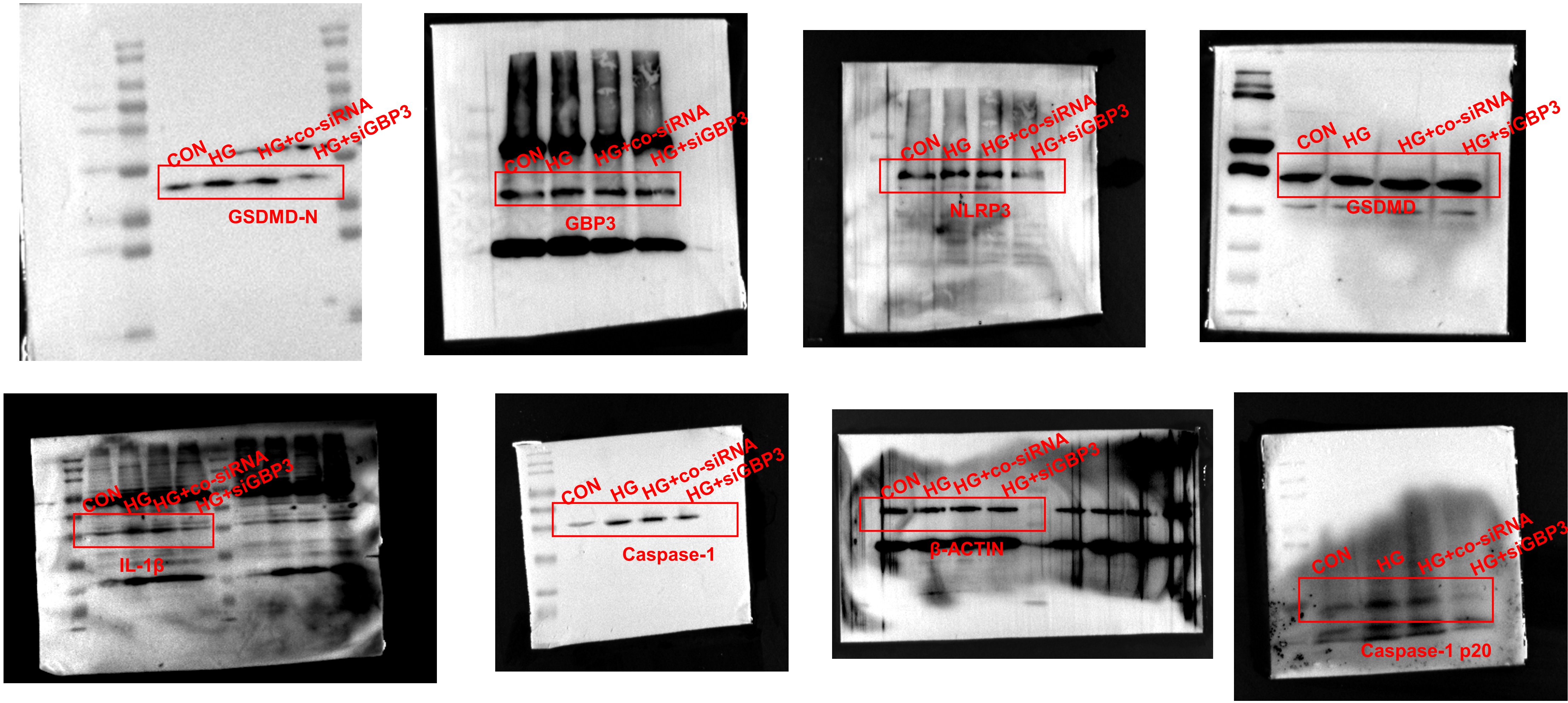
CON
HG
HG+co-siRNA
HG+siGBP3
GSDMD-N
GBP3
NLRP3
GSDMD
IL-1β
Caspase-1
β-ACTIN
Caspase-1 p20

Fig8. A

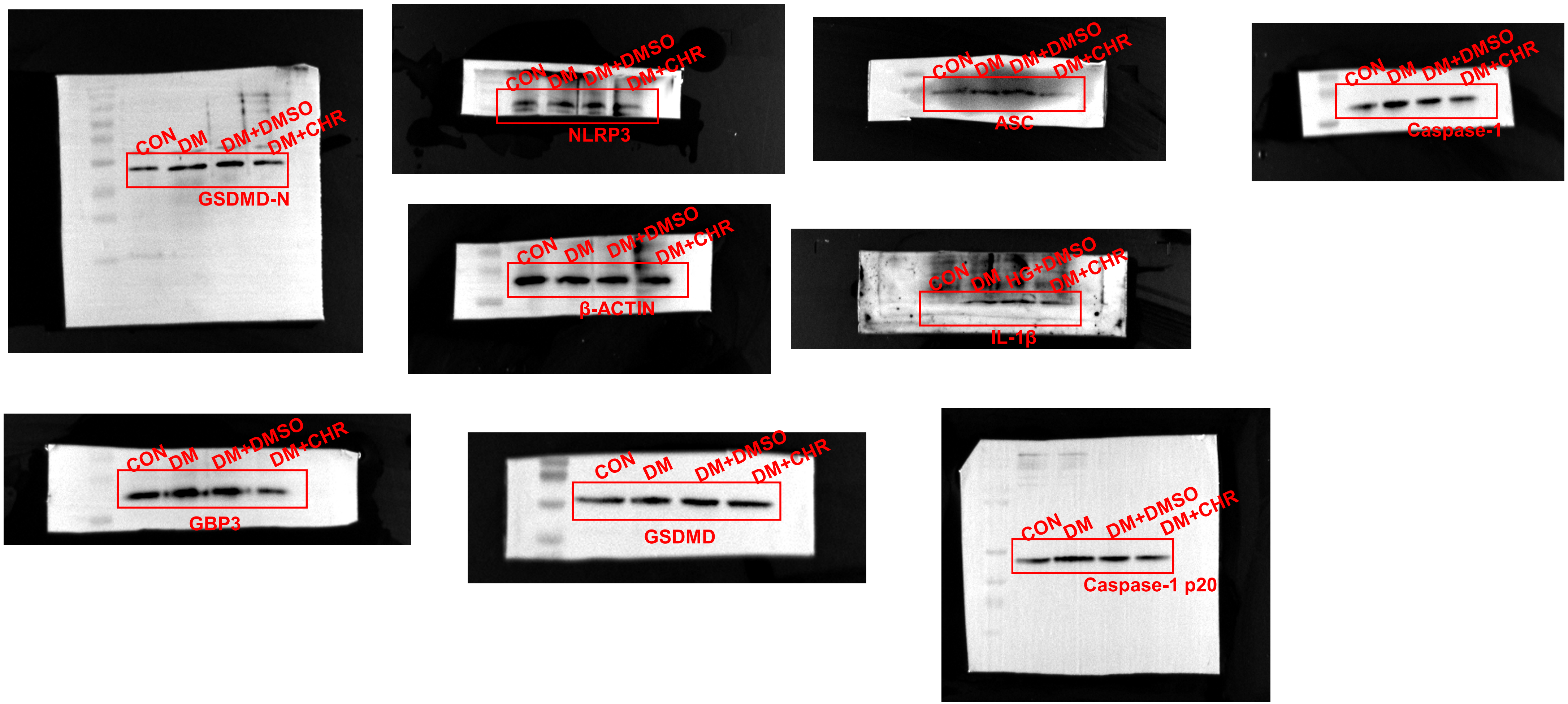
CON DM DM+DMSO DM+CHR
GSDMD-N
CON DM DM+DMSO DM+CHR
NLRP3
CON DM DM+DMSO DM+CHR
ASC
CON DM DM+DMSO DM+CHR
Caspase-1
CON DM DM+DMSO DM+CHR
β-ACTIN
CON DM HG+DMSO DM+CHR
IL-1β
CON DM DM+DMSO DM+CHR
GBP3
CON DM DM+DMSO DM+CHR
GSDMD
CON DM DM+DMSO DM+CHR
Caspase-1 p20
