# Supplementary material for: Chrysin attenuates diabetes‐induced retinal injury by inhibiting microglial GBP3/NLRP3/GSDMD‐mediated pyroptosis
Source: Animal Model Exp Med. 2026 Sep 24;9(10):2030–43. doi: 10.1002/ame2.70293 (PMC13602009; doi:10.1002/ame2.70293)
Supplement: Supplementary file 2 [file AME2-9-2030-s002.docx]

Supplementary Table 1. Primers Used for Quantitative RT-PCR.

| **Gene** | Forward (5´-3´) | Reverse (5´-3´) |
| --- | --- | --- |
| *Gsdmd*(mouse) | AGCAGAGGCGATCTCATTCC | GAGCCAAAACACTCCGGTTC |
| *Gapdh* (mouse) | ACCCAGAAGACTGTGGATGG | CACATTGGGGGTAGGAACAC |
| *Asc*(mouse) | GACAGTGCAACTGCGAGAAG | CGACTCCAGATAGTAGCTGACAA |
| *Nlrp3*(mouse) | TGACCTCTGTGCTCAAAACCA | AGTTTACAGTCCGGGTGCAG |
| *Caspase-1*(mouse) | AAGAATTTGCTGCCTGCCCA | CCACGGCATGCCTGAATAATG |
| *Il-1β* (mouse) | TGAAATGCCACCTTTTGACAGTG | ATGTGCTGCTGCGAGATTTG |
| *Gbp3*(mouse) | CTGACAGTAAATCTGGAAGCCAT | CCGTCCTGCAAGACGATTCA |
| *Gbp2*(mouse) | TGGGGTAGACGATTCCGCTAA | AGAAGTGACGGGTTTTCCGTT |
| *Gbp7*(mouse) | AACTGAGGGTGAACTCCAAAGC | GTTTCAGACCTAACTGTGGTGC |
